# Identification of the RGG Box Motif in Shadoo: RNA-Binding and Signaling Roles?

**DOI:** 10.4137/bbi.s1075

**Published:** 2008-11-19

**Authors:** Susan M. Corley, Jill E. Gready

**Affiliations:** Computational Proteomics and Therapy Design Group, Division of Molecular Bioscience, John Curtin School of Medical Research, Australian National University, PO Box 334, Canberra ACT 2601 Australia

**Keywords:** prion protein, RGG motif, RNA-binding protein, Shadoo, comparative genomics, conceptual biology, methylation, phosphorylation

## Abstract

Using comparative genomics and *in-silico* analyses, we previously identified a new member of the prion-protein (PrP) family, the gene *SPRN*, encoding the protein Shadoo (Sho), and suggested its functions might overlap with those of PrP. Extended bioinformatics and conceptual biology studies to elucidate Sho’s functions now reveal Sho has a conserved RGG-box motif, a well-known RNA-binding motif characterized in proteins such as FragileX Mental Retardation Protein. We report a systematic comparative analysis of RGG-box containing proteins which highlights the motif’s functional versatility and supports the suggestion that Sho plays a dual role in cell signaling and RNA binding in brain. These findings provide a further link to PrP, which has well-characterized RNA-binding properties.

## Introduction

In 2003 we discovered a new gene, *SPRN*, which codes for a 151-residue protein (including N- and C-terminal signal sequences) with topographical similarities unique to prion protein (PrP), and which is highly conserved between fish and mammals (for an analysis of the similarities between PrP and Sho see Premzl et al. 2003). We called this new protein Shadoo (Sho; shadow of prion protein). Like PrP, Sho is most abundant in brain (Premzl et al. 2003; Uboldi et al. 2006; Watts et al. 2007). Although the functions of Sho are as yet little characterized, it has been shown by gain and loss of function experiments (RNAi and overexpression) to be essential for CNS development in zebrafish (L. Sangiorgio, University of Milan, pers. comm.) and may have a neuroprotective effect similar to PrP (Watts et al. 2007). While PrP is notorious for its association with the transmissible spongiform encephalopathies such as Creutzfeldt Jacob Disease and Bovine Spongiform Encephelopathy (Mad Cow Disease) it has become clear in recent years that PrP has a range of normal functions, including in neurogenesis and neural plasticity (Kanaani et al. 2005; Moya et al. 2005; Santuccione et al. 2005; Steele et al. 2006). In defining the natural functions of Sho we are investigating links of the protein’s properties to those of other proteins, including PrP.

Here we report our finding that Sho has a conserved ‘RGG-box’ motif (Kiledjian and Dreyfuss, 1992) defined as a sequence of closely spaced Arg-Gly-Gly (RGG) repeats interspersed with other, often aromatic, amino acids. The RGG box proteins are one class of RNA-binding proteins (RBPs) involved in various aspects of RNA processing, including splicing, stabilizing, transport and translation of mRNAs (Burd and Dreyfuss, 1994). In addition to being an RNA-binding motif, the RGG box of some proteins is known to mediate interactions with other proteins; for a recent detailed example see Lukasiewicz et al. (2007).

The capacity to bind RNA constitutes another point of similarity with PrP which is known to bind RNA and DNA (Grossman et al. 2003). While it has been established that PrP is competent to bind nucleic acid, it is also known to bind many other ligands including polyanionic glycosaminoglycans (‘GAGs’). Given its propensity to bind polyanions, it is currently unclear whether the binding of nucleic acids is biologically relevant, that is, whether a normal function of PrP involves this type of interaction or whether binding observed experimentally may be a non-specific interaction. However, others have observed that PrP modifies DNA structure in a manner similar to proteins involved in transcriptional regulation (Bera et al. 2007) and have queried whether PrP may be involved in the biogenesis or transport of nucleic acid (Lima et al. 2006).

The approach we have used here is underpinned by a novel combination of comparative genomics (Hedges and Kumar, 2002) and conceptual biology (Blagosklonny and Pardee, 2002). By comparing Sho sequences from species ranging from fish to human and integrating these results with those of a comprehensive analysis of published sequence data and experimental findings, we have been able to put our observations into the broader context of RGG-box proteins. This has allowed us to formulate functional hypotheses for Sho.

## Materials and Methods

The amino acid sequences of 12 Sho proteins ranging from fish to human were used in this study. Ten of these sequences are available from GenBank [*Homo sapiens* Np_001012526, *Canis lupus familiaris* CAJ43798, *Bos taurus* CAJ43799, *Mus musculus* NP_898970, *Monodelphis domestica* CAF43800, *Gallus gallus* CAJ43796, *Xenopus tropicalis* CAJ43801, *Danio rerio* CAD35503, *Takifugu rubripes* CAG34291, *Tetraodon nigroviridis* CAG30521]. The sequences for *Ornithorhynchus anatinus* (platypus), *M. domestica* (American opossum), *G. gallus* (chicken), and *X. tropicalis* were initially extracted from the genomic databases (*N. Chakka*, unpublished work of this group). The sequences for *X. tropicalis* and *X. laevis* were also verified experimentally (*T. Vassilieva* and *N. Chakka*, unpublished work of this group). The sequences were aligned using ClustalW (Chenna et al. 2003). Subsequent manual adjustments in the N-terminal region were made to the alignment.

The Swiss-Prot protein database was searched using the program Prosite (Hofmann et al. 1999) http://au.expasy.org/ for known motifs within the Sho sequences. We also searched Swiss-Prot for all proteins that have an RGG-box motif, which we defined as being a sequence of at least 3 RGG repeats with no more than 6 residues between the repeats. This search produced 10 archaeal, 229 bacterial, 14 viral and 1632 eukaryotic sequences, within which there are 607 fungal, 300 plant and 70 human sequences. Examination of the human sequences showed that some well-known RGG-box proteins had not been picked up by this search. The search was then broadened to include proteins with 2 RGG repeats separated by 9, 8, 7, 6 or 5 residues. The results were visually inspected and those proteins with at least one ‘RG’ between the RGG repeats were included in our list. All uncharacterized proteins or redundant sequences were excluded. The remaining human proteins are collected in Table S1. We have only recorded the sequence beginning and ending with an RGG repeat. It should be noted that the functional RGG box may extend beyond the sequence denoted in Table S1. The RGG sequences were subsequently aligned using ClustalW.

## Results and Discussion

### Sho—RGG box

**Figure 1:**
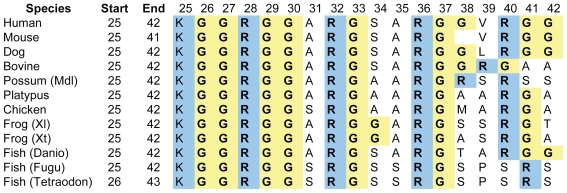
Alignment of the RGG-box sequence at the N-terminal end of Shos from fish to mammals. LHS are sequence numbers. Mdl, *Monodelphis domestica*; Xl, *Xenopus laevis*; Xt, *Xenopus tropicalis*; Danio, *Danio rerio*; Fugu, *Fugu rubripes*, Tetraodon, *Tetraodon nigroviridis*. Note that region starts with completely conserved KGG triplet. Complete RGG triplets are bolded.

A sequence alignment of the N-terminal segment from residue 25 to 42 (the mature protein starts at residue 24) of Shos from different species (Fig. 1) reveals a strictly conserved arginine methylation site (GGRGG) (Lee and Bedford, 2002) at the beginning of a cluster of RGG repeats. In Shos from human and most other Eutherian mammals there are three RGG repeats, with the first and third separated by 9 residues (**RGG**ARGSA**RGG**V**RGG)**. Thus, the RGG box of human Sho consists of 15 residues: 4 positively charged Arg residues, 7 Gly residues—6 of them dipeptides ‘GG’ which give the sequence a large degree of flexibility–, and 4 small intervening residues. This pattern diverges slightly in other species; the first RGG repeat is conserved but the second and third RGG repeats are truncated to RG in some cases. In summary, although there is some variability in the number of Gly residues in the Sho RGG box, for species from fish to human there is conservation of Lys25 and the following 3 Arg residues which are regularly spaced with 3 intervening residues between each Arg. The increased prevalence of Gly-Gly dipeptides in the higher Eutherian mammals could suggest evolutionary pressure for increased flexibility in this domain.

### Comparative analysis of RGG-box proteins—structure and composition

**Figure 2:**
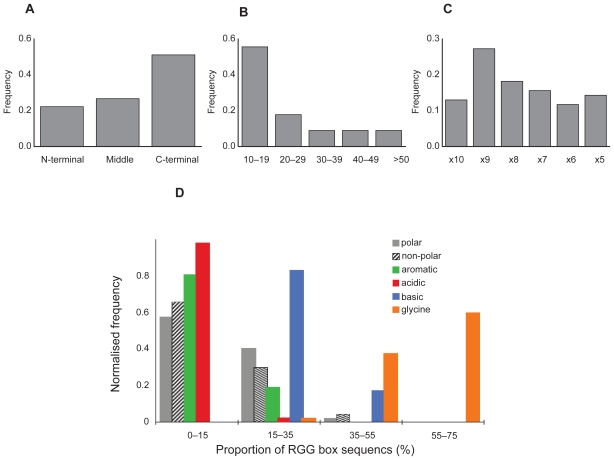
Frequency histograms of structural and compositional features of the 45 RGG sequences surveyed (Table S1). **A)** Position of the RGG box region in the proteins. N-terminal (within the first 35% of the protein sequence), C-terminal (last 35% of the protein), Middle, region between. **B)** Length of the RGG box. **C)** Spacing between RGG repeats where X may be any residue including Arg and Gly. **D)** Amino acid composition in terms of type: basic (R, K, H); acidic (D, E); Gly: aromatic (F, Y, W); non-polar amino acids (A, V, L, I, M, P) and polar (S, T, N, Q, C).

Proteins with an RGG-box motif, as defined for the purpose of this study (Methods), are presented in Supplementary Information Table S1. Most (#2–#34) are known to have an RNA-binding function. The subset of proteins highlighted in this paper is presented in Table 1. Analysis of all the proteins listed in Table S1 reveals that the RGG box is generally found at the end of the protein sequence, particularly at the C-terminus (Fig. 2A) and is mostly 10–19 residues in length (Fig. 2B). We found a slight preference for RGG repeats to be separated by 9 intervening residues (RGG-X9-RGG), as in Sho, but overall the spacing is variable (Fig. 2C).

The amino acid composition of the sequences was analysed by calculating the proportion of basic (Arg, Lys and His), acidic (Glu and Asp), aromatic (Phe, Trp and Tyr), polar (Ser, Thr, Asn, Gln and Cys), Gly and the other non-polar amino acids (Ala, Val, Leu, Ile, Met and Pro) which make up each sequence and then producing a frequency distribution for the entire set of proteins (Fig. 2D). As expected, a majority of sequences is Gly rich, with peaks in frequency at 50%–60% Gly composition while basic residues peak at 20%–30%. Although a significant number of sequences do not contain an aromatic acid between the RGG repeats, it is possible that there are aromatic residues in close sequence or spatial proximity to this domain. Very few sequences contain acidic residues.

**Figure 3:**
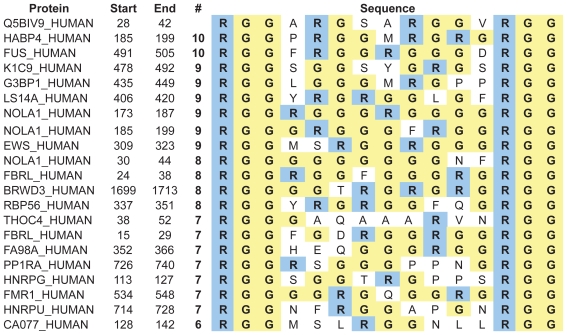
Alignment of the RGG box of proteins with RGG-X9-RGG spacing. The number of the residue at the start and end of the sequence is given, as well as the total number of exact residue matches (#) to Sho.

The Sho RGG sequence conforms to these general structural and compositional parameters. It is found at the end of the protein (N-terminus), is 15 residues long and is comprised of 47% Gly, 27% basic, 20% non-polar and 7% polar residues, and has no acidic or aromatic residues. We aligned the RGG sequence of Sho against other sequences with RGG-X9-RGG spacing in order to identify those most similar to Sho (Fig. 3). Several sequences have 50% or more residues identical to those in the Sho RGG box. Experimental studies have demonstrated that the Fragile X Mental Retardation Protein (FMRP) (#32, Table S1) (Zanotti et al. 2006) and the Herpes Simplex protein ICP27 (Mears and Rice, 1996) bind RNA with their RGG boxes which, like Sho, consist of 2 RGG repeats separated by 9 residues.

Overall, our comparative analysis supports the prediction that the RGG box of Sho is competent to bind RNA.

### Sho—predicted arginine methylation and phosphorylation sites

The Arg methylation site in Sho is completely conserved in all species from fish to human, suggesting functional importance. Arginine methylation is a common post-translational modification in RGG-box domains (Liu and Dreyfuss, 1995) which affects protein-protein interactions (Boisvert et al. 2005) and RNA binding (Dolzhanskaya et al. 2006). It influences diverse cellular processes, including cellular location of proteins (Passos et al. 2006) transcription, processing and transport of mRNAs (Yu et al. 2004) and signaling pathways (Boisvert et al. 2005).

Phosphorylation is another common post-translational modification found in RBPs. Methylation and phosphorylation mechanisms co-regulate a number of RGG-box proteins, possibly including Sho; again for a detailed example see Lukasiewicz et al. (2007). We identified 3 potential protein kinase C (PKC) phosphorylation sites (SAR (34–36 huSho), SLR (63–65 huSho) and SYR (119–121 huSho)) for Sho. One of these, SAR34–36, is within the RGG box and is found in all the Eutherian mammal sequences analysed (Fig. S1 in Supplementary Information). Phosphorylation of Ser34 would have a direct affect on the structure of the RGG box and most likely affect its function. Although the phosphorylation-site motifs are patterns with a high probability of random occurrence it is interesting to note that the presence of at least one phosphorylation site has been experimentally confirmed in 70% of the RGG-box proteins surveyed (Table S2 in Supplementary Information). This is a high proportion even taking into account the over-representation of nuclear proteins in the phosphoproteome (Olsen et al. 2006) and leads us to suggest that phosphorylation is particularly prevalent in RGG-box proteins. The finding of potential methylation and phosphorylation sites in Sho is another point of similarity with other RGG-box proteins. The existence of phosphorylation sites within Sho raises the possibility that Sho may be involved in a signaling pathway that is regulated by phosphorylation.

### Functional significance

Sho differs from most of the other proteins surveyed in that it has no other RNA-binding motifs. This is unusual but not unique as hnRNP U (#12; Table S1) has no other RNA-binding motif apart from the RGG box. The RGG box is typically associated with binding to single-stranded nucleic acids, (Zhang and Grosse, 1997) whereas additional RNA-binding motifs may allow binding of a broader range of RNA targets as is the case for nucleolin (#33, Table S1) (Ghisolfi et al. 1992). The inherent flexibility of the RGG box (Ramos et al. 2003) can also enable binding to several RNA targets, as has been shown for FMRP (Darnell et al. 2004) which binds to many RNA targets but an affinity for RNA that forms a stable G-quartet structure (Menon and Mihailescu, 2007; Ramos et al. 2003). As Sho lacks other RNA-binding motifs, we expect it to bind single-stranded nucleic acid, and potentially a range of such targets, as for FMRP.

The RGG box is a positively charged domain known to interact electrostatically with other proteins and anionic molecules. A well-characterized example is the RGG box of the yeast protein Npl3p which docks with the kinase Sky1 (Lukasiewicz et al. 2007). A non-protein example is provided by the intracellular hyaluronan binding protein (HAPB4) (#21, Table S1) which has high sequence similarity to Sho (Fig. 3). The RGG domain of HAPB4 also constitutes a glycosaminoglycan (‘GAG’) binding motif (R/K–X(7)-R/K) (Yang et al. 1994) and has been found to bind strongly and specifically to hyaluronan and weakly to RNA (Huang et al. 2000). Although it is not surprising to find this motif in an Arg-rich sequence (in fact it is present in most of the proteins included in Table S1), it has particular relevance in the case of Sho, given its cellular location.

The cellular location of Sho will determine its opportunities to bind RNA and whether this is its primary function. We originally predicted Sho to be a GPI-anchored protein (Premzl et al. 2003). This has now been confirmed in mouse (Watts et al. 2007) and for a Sho-like protein (Sho2) (Premzl et al. 2004; Strumbo et al. 2006) in zebrafish (Miesbauer et al. 2006). However, some GPI-anchored proteins, including PrP, undergo anchor cleavage (‘shedding’), (Parkin et al. 2004; Zhang et al. 2005) resulting in formation of soluble proteins which can relocate to other cellular destinations and are capable of performing multiple functions (Campana et al. 2005). While the cell surface is one likely location for Sho, it may be a multifunctional protein found in other cellular locations as well, as for PrP. If Sho sheds its GPI anchor or undergoes proteolytic cleavage before attachment to the cell membrane (Watts et al. 2007), the RGG-box domain would be available for functional roles intracellularly. Other RGG-box proteins are known to have multiple cellular locations, for example, nucleolin and the Ewing Sarcoma (EWS) protein (#26, Table S1) are found on the cell surface as well as in the nucleus and cytoplasm. In fact, there is growing evidence that some RNA-binding proteins have additional roles as cell surface receptors (Bajenova et al. 2003; Belyanskaya et al. 2003; Hirano et al. 2005) and in signaling pathways as noted for the ras GTPase activating protein binding protein 1 (Kennedy et al. 2001).

Attached to the cell surface, Sho would be positioned to act as a receptor for ligands found at the cell surface, including nucleic acids, as suggested for EWS (Belyanskaya et al. 2001). Sho may, therefore, have a role in cell signaling, similar to PrP which binds the neural cell adhesion molecule and thus participates in the tyrosine kinase fyn signaling pathway leading to neurite outgrowth (Santuccione et al. 2005). Alternatively, in this location Sho may bind other anionic ligands such as the GAG, hyaluronan, which is known to bind another GPI-anchored protein, brevican, and is involved in the structural plasticity of neural tissue (Rauch, 2004). It is interesting to note that PrP also binds GAGs including hyaluronan and heparin (Pan et al. 2002) and that GAGs may facilitate the conversion of the normal cellular PrP to the isoform found in prion disease (Yin et al. 2007).

If Sho were to shed its GPI anchor and re-enter the cell or if a segment of the N-terminal region incorporating the RGG domain was cleaved off prior to expression at the cell surface, the RGG box would be available to interact with cellular RNA. Indeed, as a small protein of no more than 123 residues, Sho would be capable of diffusing in and out of the nucleus (Cyert, 2001) and shuttling RNA from the nucleus to the cytoplasm. This is a function normally performed by RNA-binding proteins involved in neural plasticity, which participate in the biogenesis of mRNA, its transport to dendrites and repression of translation pending appropriate neural stimulation (Ule and Darnell, 2006).

## Conclusion

In summary, we have observed that Sho has a conserved RGG-box domain with similar composition to other known RGG-box proteins. We predict that this domain has functional significance and may mediate some of the neural functions already indicated for Sho. Our analysis leads us to postulate that Sho is an RNA-binding protein which may also play a role in cell signaling. Our initial experiments to test the prediction have shown Sho RGG box peptide is competent to bind RNA but further work is required to characterize the interaction.

The discovery of the RGG box in Sho opens new avenues for investigating its function and potential functional overlap with PrP. It is known that PrP plays a role in neural plasticity through its involvement in neural signaling pathways. Here we suggest that Sho may bind mRNA directly and thus play a role in neural plasticity similar to other neural RBPs.

## Supplementary Information

**Table S1:** Proteins with RGG-box domains Selected on Criteria explained in Methods.

	Database name, name (ID)	# AA	RGG domain (residue numbers)	Other RNA binding motifs^a^	Functions/Comments	R^d^/P^e^
1	SHO_HUMAN, Shadoo (Q5BIV9)	151	RGGARGSARGGVRGG (28–42)		PrP family member. Likely attached to cell membrane by GPI anchor (Premzl et al. 2003).	
2	ROA0_HUMAN, hnRNP A0 (Q13151)	305	RGGNFSGRGGFGGSRGG (192–202)	2 RRM^b^	Found in splicesome C; expected involvement in splicing, pre-mRNA processing. Similar to hnRNP A/B but less abundant. Component of ribonucleosomes. (Jurica et al. 2002).	R
3	ROA1_HUMAN, hnRNP Al (P09651)	372	RGGNFSGRGGFGGSRGG (218–234)	2 RRM	Found in splicesome C; expected involvement in splicing, pre-mRNA processing. Transport of poly (A) mRNA from nucleus to cytoplasm. (Biamonti et al. 1989; Jurica et al. 2002; Siomi and Dreyfuss, 1995).	R
4	ROA2_HUMAN, hnRNP A2 (P22626)	353	RGGNFGFGDSRGGGGNFGPGPGSNFRGG (203–230)	2 RRM	Found in splicesome C; expected involvement in splicing, pre-mRNA processing. Trafficking of RNAs containing the cis-acting A2 response element (A2RE). (Jurica et al. 2002).	R
5	ROA3_HUMAN hnRNP A3 (P51991)	378	RGGGSGNFMGRGGNFGGGGGNFGRGG (216–241)	2 RRM	Found in splicesome C; expected involvement in splicing, pre-mRNA processing. Trafficking of RNAs containing the cis-acting A2 response element (A2RE). (Jurica et al. 2002).	R
6	HNRPD_HUMAN, hnRNPD0 (Q14103)	355	RGGFAGRARGRGG (272–284)	2 RRM	Binds to mRNA with AU-rich elements (AREs) in 3′-UTR. Transcription regulator; binds to ds and ss DNA sequences. Possibly Involved in translationally coupled mRNA turnover. (Kajita et al. 1995; Tay et al. 1992).	R
7	HNRPG_HUMAN, hnRNP G (P38159)	391	RGGSGGTRGPPSRGG (113–126)	1 RRM	Found in splicesome C; expected involvement in splicing, pre-mRNA processing. (Jurica et al. 2002).	R
8			RGGGRGGSRSDRGG (373–386)			
9	HNRPK, hnRNP K (P61978)	463	RGGFDRMPPGRGGRPMPPSRRDYDDMSPRRGPPPPPPGRGGRGGSRARNLPLPPPPPPRGG (258–318)	3 KH^C^	Found in splicesome C; expected involvement in splicing, pre-mRNA processing. Major poly(C) RNA binding hnRNP. Also binds poly(C) ssDNA. (Jurica et al. 2002).	R
10	HNRPQ_HUMAN, hnRNP Q (060506)	623	RGGPGSARGVRGARGGAQQQRGRGVRGARGGRGG (526–559)	3 RRM	3 isoforms. Pre-mRNA processing. Associated with splicing intermediates and mature mRNA. Interacts preferentially with poly(A) and poly (U) RNA sequences. Region 518–549 (RGRAGYSQRGGPGSARGVRGAR GGAQQQRGRG) sufficient to bind RNA. (Mourelatos et al. 2001).	R
11	HNRPR_HUMAN, hnRNP R (043390)	633	RGGRGGPAQQQRGRGSRGSRGNRGG (543–567)	3 RRM	Found in splicesome C; expected involvement in splicing, pre-mRNA processing. (Hassfeld et al. 1998; Jurica et al. 2002).	R
12	HNRPU_HUMAN, hnRNP U (Q00839)	824	RGGGHRGRGGFNMRGGNFRGGAPGNRGG (701–728)		First discovered that hnRNP U contains a 26-residue peptide (M**RGG**NF**RGG**APGN-**RGG**YNRRG N) sufficient to account for its RNA-binding activity. This novel RNA-binding motif was defined as the RGG box. Found in splicesome C; expected involvement in splicing, pre-mRNA processing. Stabilizes specific mRNAs. Aka SAF-A; has high affinity for DNA with scaffold attachment regions (SAR). (Fackelmayer and Richter, 1994; Helbig and Fackelmayer, 2003; Jurica et al. 2002; Kiledjian and Dreyfuss, 1992; Yugami et al. 2007).	R
13	HNRL1, hnRNP U like protein 1 (Q9BUJ2)	856	RGGGGFRGRGGGGGFQRYENRGPPGGNRGGFQNRGGGSGGGGNYRGG (612–658)		Pre-mRNA processing and transport. Binds poly(G) and poly(C) RNA. Represses transcription driven by viral and cellular promoters. Associated with RBRD7, activates transcription. (Gabler et al. 1998).	R
14	PURG_HUMAN, Purine-rich element-binding protein gamma (Q9UJV8)	347	RGGGGGRGRGG (7–17)		In purine-rich element binding protein family (PUR). Binds ssDNA and RNA. Highly expressed in many tumor lines. (Liu and Johnson, 2002).	R
15	DDX4_HUMAN, DEAD box protein 4 (Q9NQI0)	724	RGGRGSFRGCRGG (147–159)		In DEAD box helicase family. Helicase activity, RNA unwinding, needed in splicing, ribosome biogenesis and RNA degradation. (Castrillon et al. 2000; Luking et al. 1998).	R
16	THOC4_HUMAN, Tho complex subunit 4 (Q86V81)	257	RGGGAQAAARVNRGG (38–52)	1 RRM	In THO/TREX complex, promotes transcriptional activation, recruited to RNA polymerase during elongation. Associated with spliced mRNA; roles in mRNA export and decay. May mediate interactions of proteins and/or RNA. (Strasser et al. 2002; Virbasius et al. 1999).	R/P
17	NOLA1_HUMAN, Nucleolar protein family A member 1 (Q9NY12)	217	RGGGRGGFNRGGGGGGFNRGGSSNHFRGGGGGGGGGNFRGGGRGGFGRGGGRGG (4–57)		Aka GAR1. Required for ribosome biogenesis and telomere maintenance. Processing or intranuclear trafficking of TERC, the RNA component of the telomerase reverse transcriptase (TERT). RGG box accessory to RNA binding. Interaction with SMN1 requires at least one of the RGG-box regions. (Bagni and Lapeyre, 1998; Whitehead et al. 2002).	R
18			RGGGRGGRGGGRGGGGRGGGRGGGFRGGRGGGGGGFRGGRGG (169–210)			
19	SFPQ_HUMAN, Splicing factor proline- and glutamine-rich (P23246)	707	RGGGGGGFHRRGGGGGRGG (9–27)	2 RRM	Pre-mRNA splicing factor. Binds to intronic polypyrimidine tracts. Possible role in nuclear retention of defective RNAs. Regulates basal and cAMP-dependent transcription. (Patton et al. 1993).	R
20	FBRL_HUMAN, Fibrillarin (P22087)	321	RGGGFGGRGGFGDRGGRGGRGGFGGGRGRGGGFRGRGRGG (8–47)		Involved in pre-rRNA processing. Component of box C/D small nucleolar ribonucleoprotein (snoRNP) particles. (Aris and Blobel, 1991; Jansen et al. 1991).	R
21	HABP4_HUMAN, Hyaluronan binding protein (HAPB4, Ki-1/57) (Q5JVS0)	413	RGGPRGGMRGRGRGG (185–199)		This sequence also constitutes a hyaluronan binding motif, (R/K-X(7)-R/K) where X is not acidic (Yang et al. 1994). This domain within HAPB4 has been found to bind strongly and specifically to hyaluronan and weakly to RNA. Involved in mRNA transport, chromatin remodeling, regulation of transcription. Interacts with chromodomain DNA helicase binding protein 3(CHD3). (Kobarg et al. 1997; Lemos et al. 2003; Passos et al. 2006).	R/P
22	PAIRB_HUMAN, Plasminogen activator inhibitor 1 RNA binding protein (Q8NC51)	408	RGGRGGRGGRGRGG (367–380)		Aka CG1–55. Regulation of mRNA stability/decay. Interacts with CHD3, similar to HABP4. (Lemos et al. 2003).	R/P
23	FUS_HUMAN, RNA binding protein FUS (P35637)	526	RGGGRGGRGGMGGSD RGG (244–261)	1 RRM	Component of nuclear riboprotein complexes. Binds ds and ss DNA. Promotes annealing of complementary ssDNAs. (Rabbitts et al. 1993).	R/P
24			RGGGNGRGGRGRGGPMGRGG (377–396))			
25			RGGRGGYDRGGYRGRGGDRGGFRGGRGGGDRGG (473–505			
26	EWS_HUMAN, Ewing sarcoma (EWS) protein (Q01844)	656	RGGFDRGGMSRGGRGGGRGGMGSAGERGG (304–332)	1 RRM	Found on cell surface as well as in the nucleus and cytoplasm. Binds RNA. Is a transcriptional activator but this activity can be repressed by the RGG box. May be involved in pre mRNA splicing and transport.	R/P
27			RGGPGGMRGGRGGLMDRGGPGGMFRGGRGGDRGGFRGGRGMDRGGFGG GRRGG (565–617)		It has been suggested that EWS protein may act as a receptor or binding protein for ligands on the cell surface, such as nucleic acids, and thus might mediate extracellular and nuclear events. Interacts with PTK2B/FAK2 then relocates from cytoplasm to ribosomes. (Belyanskaya et al. 2003; Belyanskaya et al. 2001; Ohno et al. 1994; Plougastel et al. 1993).	
28	RB56_HUMAN, TATA-binding protein-associated factor 2N (Q92804)	592	RGGYRGRGGFQGRGG (337–351)	1 RRM	Binds RNA and ssDNA. Transcription regulation. In RNA polymerase II transcriptional multiprotein complex. Similar to EWS and FUS/TLS. (Morohoshi et al. 1996).	R/P
29			RGGGYGGDRGGGYGGDRGGGYGGDRGGYGGDRGGGYGGDRGGYGGDRGGYGGDRGGYGGDRGGYGGDRSRGGYGGDRGG (459–537)			
30	CIRPB_HUMAN, Cold-inducible RNA-binding protein (Q14011)	172	RGGSAGGRGFFRGGRGRGRGFSRGG (94–118)	1 RRM	Cold-induced suppression of cell proliferation. Activates the ERK pathway. (Nishiyama et al. 1997).	
31	PP1RA_HUMAN, Serine/threonine- protein phosphatase 1 regulatory subunit (Q96QC0)	940	RGGPGPGPGPYHRGRGGRGGNEPPPPPPPFRGARGGRSGGGPPNGRGG (693–740)		Aka p99. Binds mRNA, ssDNA, poly(A) and poly(G). Inhibits phosphatase activities when phosporylated. (Kreivi et al. 1997; Totaro et al. 1998).	R
32	FMR1_HUMAN, Fragile X Mental Retardation Protein (FRMP) (Q06787)	632	RGGGGRGQGGRGRGG (534–548)	2 KH	Binds many mRNA transcripts. Transports mRNA from nucleus to cytoplasm. Involved in neural plasticity through translational repression. (Bagni and Greenough, 2005; Darnell et al. 2001; Ule and Darnell, 2006; Zalfa et al. 2003).	R/P
33	NUCL_HUMAN, Nucleolin (P19338)	710	RGGGRGGFGGRGGGRGGRGGFGGRGRGGFGGRGGFRGGRGG (656–696)	4 RRM	Found on cell surface as well as in the nucleus and cytoplasm. RGG box is necessary for efficient RNA binding and possibly operates by unstacking RNA bases, but the RRMs are required for specific RNA recognition. Duplex DNA, ssDNA and RNA are all effective ligands for nucleolin. Associated with intranucleolar chromatin and preribosomal particles. Binds to histone HI to induce chromatin decondensation. When attached to the cell surface, nucleolin binds the proteins cytokine MK and HB-19 through its RGG box and acts as cell surface receptor. (Ghisolfi et al. 1992; Hirano et al. 2005; Said et al. 2002).	R/P
34	G3BP1_HUMAN, Ras GTPase-activating protein-binding protein 1 (Q13283)	466	RGGLGGGMRGPPRGG (435–449)	1 RRM	G3BP has a role in the ras-signaling pathway affecting cell proliferation and survival as well as being involved in RNA metabolism. Cleaves MYC mRNA And has Helicase activity—unwinds DNA/DNA, RNA/DNA and RNA/RNA. Combining these two functions, it has been suggested the G3BPs are members of a novel subclass of RNA-binding proteins which act at the level of RNA metabolism in response to cell signaling allowing the cell to rapidly control protein activity at a stage after transcription. Also involved in formation of stress granules. (Irvine et al. 2004; Kennedy et al. 2001; Tourriere et al. 2003; Tourriere et al. 2001).	R/P
35	RGMC_HUMAN, Hemojuvelin (precursor) (Q6ZVN8)	426	RGGGSSGALRGGGGGGRGG (54–72)		In repulsive guidance molecule (RGM) family; RGMa and RGMb involved in neural development. GPI anchored. Interacts with neogenin which regulates shedding of GPI anchor. Binding cytokines BMP2 and BMP4 affects BMP signaling pathway and expression of hepcidin. Function of RGG domain unknown. (Matsunaga and Chedotal, 2004; Zhang et al. 2007; Zhang et al. 2005).	P
36	ZNH14_HUMAN, Zinc finger HIT domain-containing protein 4 (Q9C086)	343	RGGRGGARGERRGG (238–251)		Aka PAPA-1. Induces growth and cell cycle arrest at Gl phase. Interacts with splicing factors altering pre-mRNA splicing. Complexes with other nucleolar proteins. (Kuroda et al. 2004; Maita et al. 2004).	P
37	K1C9_HUMAN, Keratin type 1 cytoskeletal 9 (P35527)	623	RGGSGGSYGRGSRGG (478–492)		Cytoskeletal and microfibrillar keratin. Function in mature or developing palmar and plantar skin (Langbein et al. 1993).	
38	MRE11_HUMAN, Double strand break repair protein MRE 11A (P49959)	708	RGGRGQNSASRGG (577–589)		In MRN complex, role in dsDNA repair, recombination, maintenance of telomere integrity and meiosis. (Petrini et al. 1995).	
39	WBP7_HUMAN WW domain-binding protein 7 (Q9UMN6)	2715	RGGQSSRGGRGGRGRGRGG (281–299)		WW domain-binding (Trithorax homolog 2). Possible transcriptional regulator. (FitzGerald and Diaz, 1999).	
40	BRWD3_HUMAN, Bromodomain and WD repeat-containing protein 3 (Q6RI45)	1802	RGGGGTRGRGRGRGG (1699–1713)		In WD repeat protein family involved in cell-cycle progression, signal transduction, apoptosis, gene regulation. Possible transcription factor with 2 bromodomains and 9 WD repeats. May be involved in Jak/Stat pathway. (Vodermaier, 2001).	
41	CA077_HUMAN, Uncharacterised protein Clorf77 (Q9Y3Y2)	248	RGGVRGRGGPGRGG (153–166)		NA	
42	FA98A_HUMAN, Protein FAM98A	519	RGGHEQGGGRGGRGGYDHGGRGG (352–374)		NA	
43	(Q8NCA5) FA98A_HUMAN (Q8NCA5)	519	RGGGRGGRGGRGGRGG (458–473)		NA	
44	LS14A_HUMAN, LSM14 protein homolog A (Q8ND56)	463	RGGYRGRGGLGFRGGRGRGGGRGG (406–429)		Putative alpha synuclein binding protein.	

a RNA-binding motifs in addition to the RGG box.

b RRM = 80–90 amino acid sequence containing RNP-1 (octapeptide) and RNP-2 (6 amino acid) consensus sequences.

c K homology region as in hnRNP K.

d RNA binding.

e Protein binding.

**Table S2:** Phosphorylation sites in RGG box proteins surveyed in this study^a^.

Protein	Id	PKC^b^	CK2^c^	TYR^d^	Expt^e^
SHO_HUMAN	Q5BIV9	3	0	0	
ROAO_HUMAN	Q13151	4	3	0	2
ROA1_HUMAN	P09651	10	10	0	9
ROA2_HUMAN	P22626	9	4	0	6
ROA3_HUMAN	P51991	9	7	0	6
HNRPD_HUMAN	Q14103	9	6	1	7
HNRPG_HUMAN	P38159	18	16	1	7
HNRPK	P61978	7	12	1	6
HNRPQ_HUMAN	O60506	7	3	2	2
HNRPR_HUMAN	O43390	5	5	2	
HNRPU_HUMAN	Q00839	10	5	0	5
HNRL1	Q9BUJ2	6	9	0	3
PURG_HUMAN	Q9UJV8	6	2	0	1
DDX4_HUMAN	Q9NQI0	16	14	0	
THOC4_HUMAN	Q86V81	4	5	0	1
NOLA1_HUMAN	Q9NY12	3	1	0	
SFPQ_HUMAN	P23246	7	4	2	1
FBRL_HUMAN	P22087	5	3	0	
HABP4_HUMAN	Q5JVS0	5	8	2	2
PAIRB_HUMAN	Q8NC51	6	10	0	12
FUS_HUMAN	P35637	7	6	1	
EWS_HUMAN	Q01844	4	5	0	
RB56_HUMAN	Q92804	7	12	2	1
CIRPB_HUMAN	Q14011	4	2	1	
PP1RA_HUMAN	Q96QC0	10	12	2	4
FMR1_HUMAN	Q06787	9	12	1	1^f^
NUCL_HUMAN	P19338	8	23	0	14
G3BP1_HUMAN	Q13283	2	6	1	5
RGMC_HUMAN	Q6ZVN8	11	2	0	
ZNH14_HUMAN	Q9C086	3	0	0	
K1C9_HUMAN	P35527	7	14	3	
MRE11_HUMAN	P49959	16	17	0	4
WBP7_HUMAN	Q9UMN6	40	36	3	4
BRWD3_HUMAN	Q6RI45	34	42	4	4
CA077_HUMAN	Q9Y3Y2	4	2	0	1
FA98A_HUMAN	Q8NCA5	5	9	1	
LS14A_HUMAN	Q8ND56	4	7	1	11

a Searches were conducted using the ScanProsite program available on the ExPASy Proteomics Server of the Swiss Institute of Bioinformatics website http://au.expasy.org/.

b Number of protein kinase C phosphorylation sites (PS00005).

c Number of casein kinase II phosphorylation sites (PS00006).

d Number of tyrosine kinase phosphorylation sites (PS00007).

e As annotated in the SwissProt database.

f Mazroui, R., Huot, M.E., Tremblay, S., Boilard, N., Labelle, Y. and Khandjian, E.W. (2003) Fragile X Mental Retardation protein determinants required for its association with polyribosomal mRNPs. *Hum Mol Genet,* 12;3087–96.

**Figure S1:**
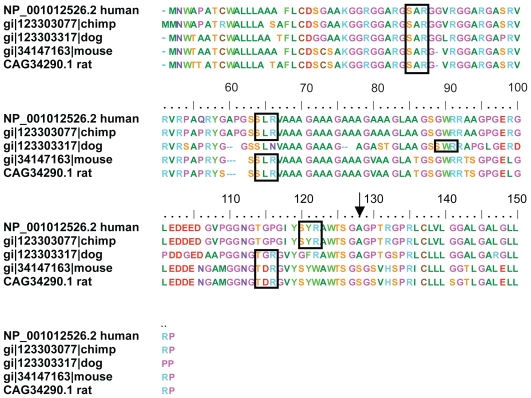
Alignment of Sho sequences of Eutherian mammals. The 3 PKC phosphorylation sites are indicated by boxes. The N-terminal and C-terminal cleavage sites are shown by arrows.

ReferencesArisJPBlobelG1991cDNA cloning and sequencing of human fibrillarin, a conserved nucleolar protein recognized by autoimmune antiseraProc. Natl. Acad. Sci. U.S.A889315184696810.1073/pnas.88.3.931PMC50928BagniCGreenoughWT2005From mRNP trafficking to spine dysmorphogenesis: the roots of fragile X syndromeNat. Rev. Neurosci6376871586118010.1038/nrn1667BagniCLapeyreB1998Garl p binds to the small nucleolar RNAs snR10 and snR30 in vitro through a nontypical RNA binding elementJ. Biol. Chem2731086873955656110.1074/jbc.273.18.10868BelyanskayaLLDelattreOGehringH2003Expression and subcellular localization of Ewing sarcoma (EWS) protein is affected by the methylation processExp. Cell. Res288374811291512810.1016/s0014-4827(03)00221-0BelyanskayaLLGehrigPMGehringH2001Exposure on cell surface and extensive arginine methylation of ewing sarcoma (EWS) proteinJ. Biol. Chem2761868171127890610.1074/jbc.M011446200BiamontiGBuvoliMBassiMTMorandiCCobianchiFRivaS1989Isolation of an active gene encoding human hnRNP protein Al. Evidence for alternative splicingJ. Mol. Biol207491503276092210.1016/0022-2836(89)90459-2CastrillonDHQuadeBJWangTYQuigleyCCrumCP2000The human VASA gene is specifically expressed in the germ cell lineageProc. Natl. Acad. Sci. U.S.A979585901092020210.1073/pnas.160274797PMC16908DarnellJCJensenKBJinPBrownVWarrenSTDarnellRB2001Fragile X mental retardation protein targets G quartet mRNAs important for neuronal functionCell107489991171918910.1016/s0092-8674(01)00566-9FackelmayerFORichterA1994hnRNP-U/SAF-A is encoded by two differentially polyadenylated mRNAs in human cellsBiochim. Biophys. Acta12172324750919510.1016/0167-4781(94)90044-2FitzGeraldKTDiazMO1999MLL2: A new mammalian member of the trx/MLL family of genesGenomics59187921040943010.1006/geno.1999.5860GablerSSchüttHGroitlPWolfHShenkTDobnerT1998E1B 55-kilodalton-associated protein: a cellular protein with RNA-binding activity implicated in nucleocytoplasmic transport of adenovirus and cellular mRNAsJ. Virol72796071973383410.1128/jvi.72.10.7960-7971.1998PMC110131GhisolfiLKharratAJosephGAmalricFErardM1992Concerted activities of the RNA recognition and the glycine-rich C-terminal domains of nucleolin are required for efficient complex formation with pre-ribosomal RNAEur. J. Biochem2095418142566010.1111/j.1432-1033.1992.tb17318.xHassfeldWChanEKMathisonDAPortmanDDreyfussGSteinerGTanEM1998Molecular definition of heterogeneous nuclear ribonucleoprotein R (hnRNP R.) using autoimmune antibody: immunological relationship with hnRNP PNucleic. Acids. Res2643945942149710.1093/nar/26.2.439PMC147279HelbigRFackelmayerFO2003Scaffold attachment factor A (SAF-A) is concentrated in inactive X chromosome territories through its RGG domainChromosoma111173821460846310.1007/s00412-003-0258-0HiranoKMikiYHiraiYSatoRItohTHayashiAYamanakaMEdaSBeppuM2005A multifunctional shuttling protein nucleolin is a macrophage receptor for apoptotic cellsJ. Biol. Chem28039284931613551710.1074/jbc.M505275200IrvineKStirlingRHumeDKennedyD2004Rasputin, more promiscuous than ever: a review of G3BPInt. J. Dev. Biol481065771560269210.1387/ijdb.041893kiJansenRPHurtECKernHLehtonenHCarmo-FonsecaMLapeyreBTollerveyD1991Evolutionary conservation of the human nucleolar protein fibrillarin and its functional expression in yeastJ. Cell. Biol11371529202664610.1083/jcb.113.4.715PMC2288999JuricaMSLickliderLJGygiSRGrigorieffNMooreMJ2002Purification and characterization of native spliceosomes suitable for three-dimensional structural analysisRna8426391199163810.1017/s1355838202021088PMC1370266KajitaYNakayamaJAizawaMIshikawaF1995The UUAG-specific RNA binding protein, heterogeneous nuclear ribonucleoprotein DO. Common modular structure and binding properties of the 2xRBD-Gly familyJ. Biol. Chem2702216775767319510.1074/jbc.270.38.22167KennedyDFrenchJGuitardERuKTocqueBMattickJ2001Characterization of G3BPs: tissue specific expression, chromosomal localisation and rasGAP(120) binding studiesJ. Cell. Biochem84173871174652610.1002/jcb.1277KiledjianMDreyfussG1992Primary structure and binding activity of the hnRNP U protein: binding RNA through RGG boxEmbo. J11265564162862510.1002/j.1460-2075.1992.tb05331.xPMC556741KobargJSchnittgerSFonatschCLemkeHBowenMABuckFHansenHP1997Characterization, mapping and partial cDNA sequence of the 57-kD intracellular Ki-1 antigenExp. Clin. Immunogenet14273809523163KreiviJPTrinkle-MulcahyLLyonCEMorriceNACohenPLamondAI1997Purification and characterisation of p99, a nuclear modulator of protein phosphatase 1 activityFEBS Lett4205762945055010.1016/s0014-5793(97)01485-3KurodaTSMaitaHTabataTTairaTKitauraHArigaHIguchi-ArigaSM2004A novel nucleolar protein, PAPA-1, induces growth arrest as a result of cell cycle arrest at the Gl phaseGene34083981555629710.1016/j.gene.2004.05.025LangbeinLHeidHWMollIFrankeWW1993Molecular characterization of the body site-specific human epidermal cytokeratin 9: cDNA cloning, amino acid sequence, and tissue specificity of gene expressionDifferentiation555771750786910.1111/j.1432-0436.1993.tb00033.xLemosTAPassosDONeryFCKobargJ2003Characterization of a new family of proteins that interact with the C-terminal region of the chromatin-remodeling factor CHD-3FEBS Lett53314201250515110.1016/s0014-5793(02)03737-7LiuHJohnsonEM2002Distinct proteins encoded by alternative transcripts of the PURG gene, located contrapodal to WRN. on chromosome 8, determined by differential termination/polyadenylationNucleic. Acids. Res302417261203482910.1093/nar/30.11.2417PMC117198LukingAStahlUSchmidtU1998The protein family of RNA helicasesCrit. Rev. Biochem. Mol. Biol3325996974767010.1080/10409239891204233MaitaHKitauraHKeenTJInglehearnCFArigaHIguchi-ArigaSM2004PAP-1, the mutated gene underlying the RP9 form of dominant retinitis pigmentosa, is a splicing factorExp. Cell. Res300283961547499410.1016/j.yexcr.2004.07.029MatsunagaEChedotalA2004Repulsive guidance molecule/neogenin: a novel ligand-receptor system playing multiple roles in neural developmentDev. Growth Differ4648161561013710.1111/j.1440-169x.2004.00768.xMorohoshiFAraiKTakahashiEITanigamiAOhkiM1996Cloning and mapping of a human RBP56 gene encoding a putative RNA binding protein similar to FUS/TLS and EWS proteinsGenomics38517895477910.1006/geno.1996.0591MourelatosZAbelLYongJKataokaNDreyfussG2001SMN interacts with a novel family of hnRNP and spliceosomal proteinsEMBO J205443521157447610.1093/emboj/20.19.5443PMC125643NishiyamaHHigashitsujiHYokoiHItohKDannoSMatsudaTFujitaJ1997Cloning and characterization of human CIRP (cold-inducible RNA-binding protein) cDNA and chromosomal assignment of the geneGene20411520943417210.1016/s0378-1119(97)00530-1OhnoTOuchidaMLeeLGatalicaZRaoVNReddyES1994The EWS gene, involved in Ewing family of tumors, malignant melanoma of soft parts and desmoplastic small round cell tumors, codes for an RNA binding protein with novel regulatory domainsOncogene93087978084618PassosDOBressanGCNeryFCKobargJ2006Ki-1/57 interacts with PRMT1 and is a substrate for arginine methylationFebs. J2733946611687961410.1111/j.1742-4658.2006.05399.xPattonJGPorroEBGalceranJTempstPNadal-GinardB1993Cloning and characterization of PSF, a novel pre-mRNA splicing factorGenes. Dev7393406844940110.1101/gad.7.3.393PetriniJHWalshMEDiMareCChenXNKorenbergJRWeaverDT1995Isolation and characterization of the human MRE11 homologueGenomics29806853010410.1006/geno.1995.1217PlougastelBZucmanJPeterMThomasGDelattreO1993Genomic structure of the EWS gene and its relationship to EWSR.1, a site of tumor-associated chromosome translocationGenomics1860915830757010.1016/s0888-7543(05)80363-5PremzlMSangiorgioLStrumboBMarshall GravesJASimonicTGreadyJE2003Shadoo, a new protein highly conserved from fish to mammals and with similarity to prion proteinGene314891021452772110.1016/s0378-1119(03)00707-8RabbittsTHForsterALarsonRNathanP1993Fusion of the dominant negative transcription regulator CHOP with a novel gene FUS by translocation t(12; 16) in malignant liposarcomaNat. Genet417580750381110.1038/ng0693-175SaidEAKrustBNisoleSSvabJBriandJPHovanessianAG2002The anti-HIV cytokine midkine binds the cell surface-expressed nucleolin as a low affinity receptorJ. Biol. Chem1113149250210.1074/jbc.M20119420012147681SiomiHDreyfussG1995A nuclear localization domain in the hnRNP Al proteinJ. Cell. Biol12955160773039510.1083/jcb.129.3.551PMC2120450StrasserKMasudaSMasonPPfannstielJOppizziMRodriguez-NavarroSRondonAGAguileraAStruhlKReedRHurtE2002TREX is a conserved complex coupling transcription with messenger RNA exportNature41730481197927710.1038/nature746TayNChanSHRenEC1992Identification and cloning of a novel heterogeneous nuclear ribonucleoprotein C-like protein that functions as a transcriptional activator of the hepatitis B. virus enhancer IIJ. Virol6668418143349710.1128/jvi.66.12.6841-6848.1992PMC240284TotaroAGrifaACarellaMRommensJMValentinoMARoettoAZelanteLGaspariniP1998Cloning of a new gene (FB.19) within HLA class I regionBiochem. Biophys. Res. Commun2505557978438110.1006/bbrc.1998.9354TourriereHChebliKZekriLCourselaudBBlanchardJMBertrandETaziJ2003The RasGAP-associated endoribonuclease G3BP assembles stress granulesJ. Cell. Biol160823311264261010.1083/jcb.200212128PMC2173781TourriereHGallouziIEChebliKCaponyJPMouaikelJvan der GeerPTaziJ2001RasGAP-associated endoribonuclease G3Bp: selective RNA degradation and phosphorylation-dependent localizationMol. Cell. Biol217747601160451010.1128/MCB.21.22.7747-7760.2001PMC99945UleJDarnellRB2006RNA binding proteins and the regulation of neuronal synaptic plasticityCurr. Opin. Neurobiol16102101641800110.1016/j.conb.2006.01.003VirbasiusCMWagnerSGreenMR1999A human nuclear-localized chaperone that regulates dimerization, DNA binding, and transcriptional activity of bZIP proteinsMol. Cell4219281048833710.1016/s1097-2765(00)80369-xVodermaierHC2001Cell. cycle: Waiters serving the Destruction machineryCurr. Biol11R834371167693910.1016/s0960-9822(01)00498-5WhiteheadSEJonesKWZhangXChengXTernsRMTernsMP2002Determinants of the interaction of the spinal muscular atrophy disease protein SMN. with the dimethylarginine-modified box H/ACA small nucleolar ribonucleoprotein GARlJ. Biol. Chem27748087931224409610.1074/jbc.M204551200YugamiMKabeYYamaguchiYWadaTHandaH2007hnRNP-U enhances the expression of specific genes by stabilizing mRNAFEBS Lett581171717430610.1016/S0014-5793(07)01283-5PMC7130276ZalfaFGiorgiMPrimeranoBMoroADi PentaAReisSOostraBBagniC2003The fragile X syndrome protein FMRP associates with BC1 RNA and regulates the translation of specific mRNAs at synapsesCell112317271258152210.1016/s0092-8674(03)00079-5ZhangASAndersonSAMeyersKRHernandezCEisensteinRSEnnsCA2007Evidence that inhibition of hemojuvelin shedding in response to iron is mediated through neogeninJ. Biol. Chem28212547561733195310.1074/jbc.M608788200ZhangASWestAPJrWymanAEBjorkmanPJEnnsCA2005Interaction of hemojuvelin with neogenin results in iron accumulation in human embryonic kidney 293 cellsJ. Biol. Chem28033885941610311710.1074/jbc.M506207200

## Figures and Tables

**Table 1:** Subset of RGG-box proteins (see Table S1 in Supplementary Information for full list).

No. (#)^a^	Name (ID)	RGG domain (residue numbers)	Other RNA-binding motifs^b^	Functions/Comments R^e^/P^f^ (For details and references see Table S1)
1	Shadoo Q5BIV9	RGGARGSARGGVRGG (28–42)		PrP family member. Likely attached to cell membrane by GPI anchor.
12	hnRNP U Q00839	RGGGHRGRGGFNMRGGNFRGGAPGNRGG (701–728)		RGG box first identified when a 26-residue sequence (M**RGG**NF**RGG**APGN**RGG**YNRRGN) found to be sufficient for RNA binding. Expected involvement in splicing, pre-mRNA processing and stabilizes specific mRNAs. **R**
21	HABP4 Hyaluronan binding protein 4 Q5JVS0	RGGPRGGMRGRGRGG (185–199)		Also constitutes a hyaluronan binding motif, (R/K–X(7)-R/K) where X is not acidic. Binds strongly and specifically to hyaluronan and weakly to RNA. Involved in mRNA transport, chromatin remodeling, regulation of transcription. **R/P**
26	EWS Ewing sarcoma Q01844	RGGFDRGGMSRGGRGGGRGGMGSAGERGG (304–332) and RGGPGGMRGGRGGLMDRGGPGGMFRGGRGGDRGGFRGGRGMDRGGFGGGRRGG (565–617)	1 RRM^c^	Found on cell surface, nucleus and cytoplasm. Is a transcriptional activator but this activity can be repressed by RGG box. May be involved in pre-mRNA splicing and transport. Suggested that EWS protein acts as a receptor or binding protein for ligands on cell surface, such as nucleic acids, and thus might mediate extracellular and nuclear events. **R/P**
32	FMRP Fragile X Mental Retardation Protein Q06787	RGGGGRGQGGRGRGG (534–548)	2 KH^d^	Binds many mRNA transcripts. Transports mRNA from nucleus to cytoplasm. Involved in neural plasticity through translational repression. **R/P**
33	Nucleolin P19338	RGGGRGGFGGRGGGRGGRGGFGGRGRGGFGGRGGFRGGRGG (656–696)	4 RRM	Found on cell surface, nucleus and cytoplasm. RGG box is necessary for efficient RNA binding but the RRMs are required for specific RNA recognition. Duplex DNA, ssDNA and RNA are all effective ligands. Acts as cell surface receptor—binds cytokine MK and HB-19 through its RGG box. **R/P**
34	G3BP1 Ras GTPase-activating protein-binding protein 1 Q13283	RGGLGGGMRGPPRGG (435–449)	1 RRM	Role in ras-signaling pathway affecting cell proliferation and survival as well as involved in RNA metabolism. Cleaves MYC mRNA and has helicase activity. Combining these functions, suggested to be member of novel sub-class of RBPs which act at level of RNA metabolism in response to cell signaling, thus allowing cell to rapidly control protein activity at a stage after transcription. **R/P**

a number of protein as appears in Table S1.

b RNA-binding motifs in addition to the RGG box.

c RRM = 80–90 amino acid sequence containing a RNP-1 (octapeptide) and RNP-2 (6 amino acid) consensus sequences.

d K homology region as in hnRNP K.

e RNA binding.

f Protein binding.
